# Biological knowledge combined with innovative engineering to reduce power plant impingement of horseshoe crabs

**DOI:** 10.1371/journal.pone.0322119

**Published:** 2025-05-05

**Authors:** Claire Crowley-McIntyre, Berlynna Heres, H. Jane Brockmann, Ryan Gandy, Lisa Gregg

**Affiliations:** 1 Florida Fish and Wildlife Conservation Commission, St. Petersburg, Florida, United States of America; 2 Department of Biology, University of Florida, Gainesville, Florida, United States of America; 3 Sarasota Bay Estuary Program, Sarasota, Florida, United States of America; University of Connecticut, UNITED STATES OF AMERICA

## Abstract

Electric generating plants (power plants) are often subject to regulatory requirements to comply with the impingement mortality reduction standards of Section 316(b) of the federal Clean Water Act. In 2014, a power plant located on the Northern Indian River Lagoon (NIRL) in Florida, identified impingement of adult American horseshoe crabs (*Limulus polyphemus,* Linneaus, hereafter; horseshoe crabs) at the intake bar screens located in the plant intake canal. In November of 2014 the plant installed a prototype fence to mitigate horseshoe crab impingement but found that an average of 50,188 horseshoe crabs per year were still being impinged. In 2015, the plant sought to collaborate with the Florida Fish and Wildlife Conservation Commission (FWC) and University of Florida to modify the fence to reduce the impingement of horseshoe crabs, while still allowing manatees access to thermal refugia and to provide unobstructed passage of sea turtles and fuel barges. The plant and collaborators worked to improve the design with consideration of the structural orientation, material composition, and natural horseshoe crab behavior. In November 2017, the plant installed the improved design, a permanently submerged deterrent wall across the width of the intake channel that achieved the goal of reducing horseshoe crab impingement while allowing the free movement of manatees, sea turtles, and fuel barges. Continued monitoring and maintenance of the intake system and deterrent wall shows horseshoe crab impingement was reduced by 97.3% to an average yearly impingement of 647.7 horseshoe crabs with final wall installation. This collaborative engineering approach illustrates the effectiveness of well-designed and maintained barriers in reducing horseshoe crab impingement while allowing compliance with regulatory changes without disruption to operations.

## Introduction

There are hundreds of power plants (including nuclear, natural gas, oil, and coal) along the East Coast of the United States, and most require water from the local environment [1]. The water is used to cool electrical-generating equipment, heated into steam that is used to spin turbines that then drive a generator that produces electricity, and used to cool steam, condensing it back into water that is then reheated and run through the turbines again. This water is drawn into the plant and either released back into the environment (once-through systems) or recirculated via closed-cycle recirculating cooling systems (e.g., cooling towers). Aquatic wildlife such as fish and invertebrates (including arthropods) can be impinged on the water intake structures of power plants [2] along with aquatic plants and debris. Often animals, such as horseshoe crabs, survive initial impingement but may experience injury or mortality when they are discarded back to the water due to physical injuries, physiological stress, and suffocation; when discarded as waste there is 100% mortality. [3]. The Environmental Protection Agency (EPA) favors the use of closed-cycle recirculating cooling systems for both new and existing power plants because this technology reduces the amount of water used, and by extension reduces entrainment and impingement of aquatic organisms. Alternatively, there are seven acceptable technologies to meet the standards for impingement mortality. Section 316(b) of the Clean Water Act, which regulates cooling water intake structures, requires that power plants that withdraw more than 2 million gallons per day (MGD) of water from waters of the United States and use at least 25 percent of the water they withdraw exclusively for cooling purposes, to use the best technology available (BTA) to minimize environmental impacts [1], including the impact on wildlife (79 FR 48299, Clean Water Act 33 U.S.C. § 1251 (1972) [4]. The implementation of these increased regulatory standards has decreased wildlife impacts overall [4], but many less-monitored species like the horseshoe crab (*Limulus polyphemus,* Linneaus; henceforth, horseshoe crab) can be overlooked and impacts can be missed due to uncertainty in regional population estimates.

The horseshoe crab is found along the entire Atlantic coast of the United States, within the Gulf of Mexico from Florida west to the Mississippi delta, and along the coasts of the Yucatan peninsula [5]. Horseshoe crabs are large, long-lived arthropods that require 9–10 years to reach sexual maturity, with a total life span of at least 20 years [6]. They are bottom feeders, generally living in shallow water eating sea worms, clams, crabs, and detritus [7]. During the spring they mate and bury their eggs in nests along beaches and shorelines [8]. Horseshoe crabs are of considerable ecological, economic, and biomedical value and are managed across much of their range [9,10]. Overall, the numbers of horseshoe crabs have declined due to habitat loss (e.g., shoreline hardening), historic overfishing, and climate change, however, strict fisheries regulations have improved population numbers in the Delaware Bay resulting in a gradual recovery in those populations [6,11].

One threat to populations is from mortality associated with impingement in power plant intakes throughout their range. Asian horseshoe crabs, including the mangrove horseshoe crab, (*Carcinoscorpius rotundicauda* Latreille) and the Indo-Pacific horseshoe crab, (*Tachypleus gigas* Müller), were impinged on the cooling water intake structure of the Kapar Power Station in Malaysia. Azila and Chong [12] found mangrove horseshoe crabs in 72% of the samples of impinged animals collected at the Kapar Power Station making it the 36^th^ most abundant of the 178 species documented [12]. Horseshoe crabs have been observed on the intake screens at Brayton Point Station in Massachusetts, a now decommissioned and demolished coal-power plant [13]. Horseshoe crabs have also been observed at Oyster Creek Nuclear Generating Station, Forked River, New Jersey (FOIA No. 2000–0098; https://www.nrc.gov/docs/ML0036/ML003698057.pdf), at Calvert Cliffs Nuclear Power Plant in Lusby, Maryland (https://www.nrc.gov/docs/ML1109/ML110970435.pdf), at the E.F. Barrett Power Generation facility at Island Park, New York (https://www.riverkeeper.org/blogs/docket/a-view-to-a-fish-kill-a-firsthand-perspective-on-fish-killing-cooling-systems/), and at the PSEG New Haven Harbor power plant in New Haven, Connecticut where large numbers of horseshoe crabs were observed in the intake forebay [7]. At the Connecticut power plants, Aquatic Organism Return (AOR) systems and narrowing of the bars on the intake racks has reduced impingement of horseshoe crabs and other species [7]. In Florida, studies at the coal- powered Cape Canaveral and Indian River power plants in 1975 and 1979 documented large numbers of horseshoe crabs impinged on an annual basis (1975: 69,662 and 104,000; 1979: 39,097 and 53,121 respectively; [10,14]).

In 2011 a conventional coal powered plant in the Northern Indian River Lagoon (NIRL) was decommissioned and replaced with a modernized combined cycle plant fueled by natural gas that began commercial operations in April 2013. During the 2014 horseshoe crab spawning season, horseshoe crabs were being impinged at the intake bar screens. The plant needed to find a solution to address horseshoe crab impingement to comply with Section 316(b) of the Clean Water Act and the Florida Department of Environmental Protection (FDEP) Conditions of Certification (Section B.IV.A.9). This manuscript reports on the process and successful solution that was reached. In 2015 a team of subject matter experts on the horseshoe crab, West Indian manatee (*Trichechus manatus* Linnaeus), and sea turtle (Cheloniidae sp.) were assembled from the Florida Fish and Wildlife Conservation Commission (FWC), the University of Florida, and the plant’s engineers and engineering consultants. The project team could find no examples of horseshoe crab impingement mitigation from other power plants to replicate, necessitating original design and testing solutions. Designing a horseshoe crab deterrent wall for this specific power plant intake canal was challenging. The biggest difficulty was to make the deterrent wall effective in keeping horseshoe crabs out, while allowing West Indian manatees and sea turtle species access to the warm water refuge area near the power plant. In addition, fuel barges had to freely access the area several times a year. These problems required a short structure (~1 m) attached to the bottom of the seafloor that would allow at least 2.4 m of water column above it to allow passage of West Indian manatees, sea turtles, and barges. Although horseshoe crabs normally walk along the bottom, they often burrow into the substrate [15,16], so the deterrent wall had to extend below the seafloor to prevent horseshoe crabs from burrowing underneath. Horseshoe crabs can also swim [16,17] and are attracted to water intakes because they follow currents [18,19,20]. Thus, the wall had to prevent them from swimming or climbing over the top while also deflecting them away from the intake flow [17]. Additionally, the angle of the wall could not pose an entrapment hazard to sea turtles. The following is a summary of the biological features of the NIRL horseshoe crab population and both the failures and successes of the engineering efforts, which can provide support for future investigators with similar problems. This innovative solution could be replicated at other power plants where horseshoe crab impingement occurs, saving horseshoe crabs, time, and money.

## Methods

### Location

**Fig 1:**
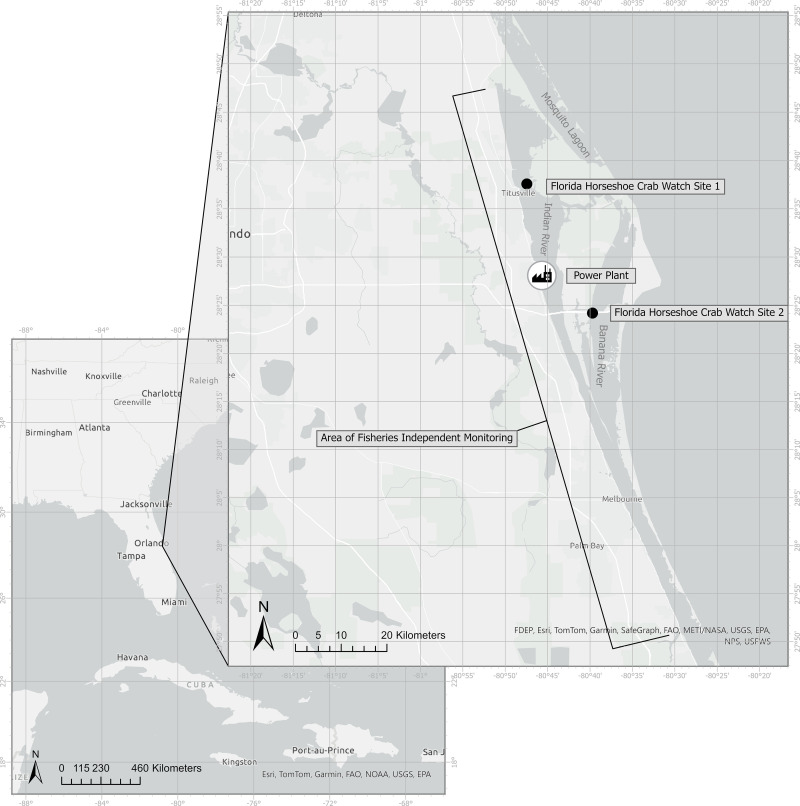
Location of the power plant, Florida Horseshoe Crab Watch sites, where spawning horseshoe crabs are surveyed during the highest tides (black circles), the sampling boundary of FWRI’s Fisheries Independent Monitoring, and the Brevard County boundaries (horizontal black lines).

The power plant is in Brevard County, within the Northern Indian River Lagoon (Fig 1). The Indian River Lagoon (IRL) is a 156-mile-long lagoon composed of three smaller lagoons, the Mosquito Lagoon, Banana River, and Indian River that span five counties on the eastern coast of Florida, United States [21]. The plant is comprised of three nominal 250 MW combustion turbines and a nominal 500 MW steam turbine operated in combined-cycle mode with a total generating capacity of 1,250 MW. The power plant is fueled by natural gas with ultra-low sulfur distillate fuel oil as a backup fuel source. The facility withdraws cooling water from the Canaveral Pool of the NIRL through the intake canal located on the western shoreline on the south side of the plant. The cooling water intake structure has two intakes, each with two 10-ft wide intake bays and two single-stage circulating water pumps within the intake canal. The four single-stage circulating water pumps are rated at 146,862 gallons per minute (gpm) each, resulting in a total combined capacity of 587,448 gpm (846 million gallons per day (mgd)). However, the design intake flow (DIF) is 634.5 mgd (440,586 gpm) with three circulating water pumps running during routine operations and one stand-by pump reserved as a backup.

### Process of deterrent structure implementation

To address the impinged horseshoe crabs in early 2014, a prototype fence was installed in November 2014. The prototype fence did not sufficiently exclude horseshoe crabs from the intake system, so the plant engaged with subject matter experts to monitor and modify the prototype fence design and eventually install a permanent deterrent wall. It was not a research project to determine the performance of each component. Rather, this approach allowed for observation and adaptation, to arrive at the design of the final deterrent wall. After the prototype fence was installed, the plant observed its attributes of location, material performance, fence height, fence angle, insertion depth in the substrate, time and maintenance costs, velocity of water movement, and fouling of the material to determine if changes were needed and guide further design modifications and monitoring. The material, location, insertion depth, fouling, and maintenance of the fence were determined to be weak points in the design and were modified in the final deterrent wall design. The prototype fence and observation of its function was essential to the design and success of the permanent deterrent wall that was installed in November of 2017.

### Prototype fence and design modification

**Fig 2:**
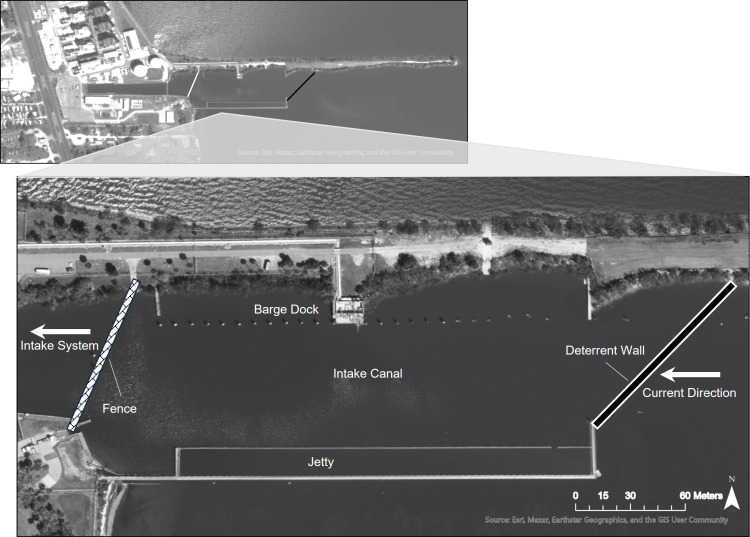
Power Plant Intake Canal and locations of the Barge Dock, Jetty, Prototype Fence (brick bar), Deterrent Wall (black solid bar). The current direction and direction to the intake system are indicated by white arrows.

**Fig 3:**
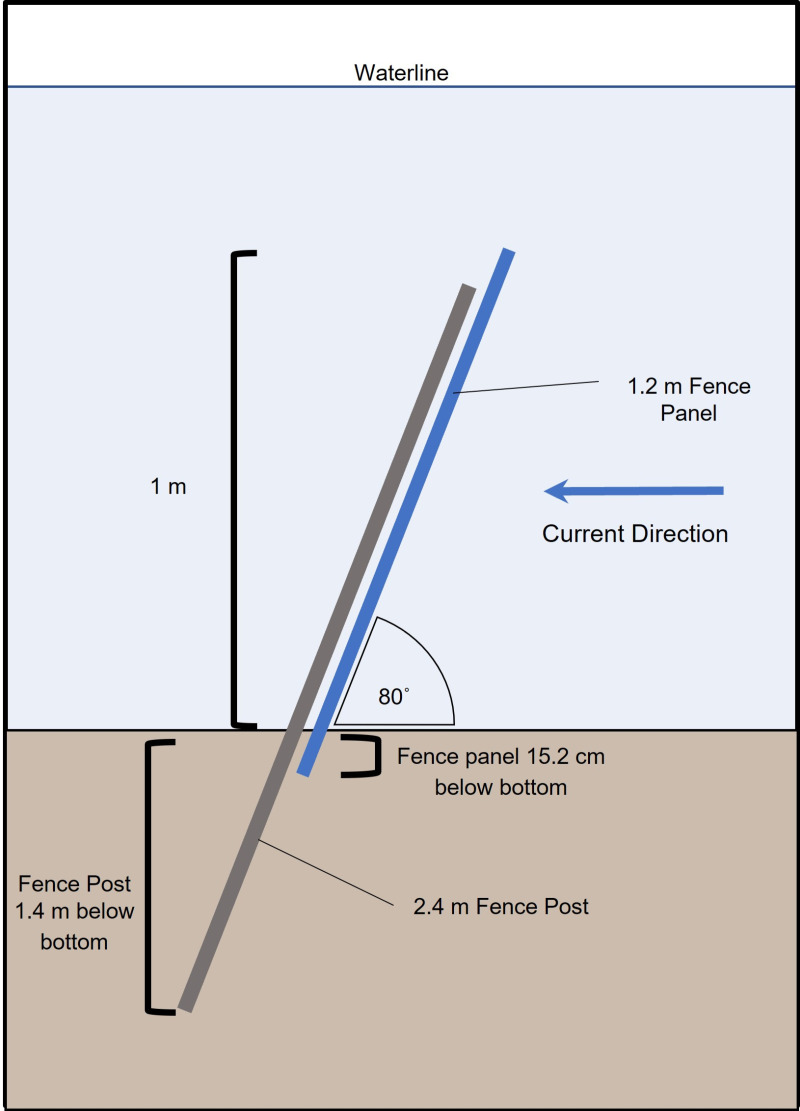
Schematic of the Prototype Fence installed in the intake canal in November 2014.

The deterrent structure had two phases: the prototype fence design phase from November 1, 2014, to October 31, 2017, and the permanent deterrent wall phase from November 1, 2017, to present (Table 1). The prototype fence installed in November 2014 was constructed of 4.88-meter-wide by 1.22-meter-high panels of 5.08 cm x 10.16 cm galvanized wire mesh, roughly 76 m across the western end of the intake channel (Figs 2 and 3). The panels were placed so they overlapped adjacent panels by 0.3 m and were attached to 2.44 m fence posts installed at an 80° angle to the seafloor. The 80° angle of the fence was oriented into the 0.46 mps current created by the intake and resulted in an acute angle for horseshoe crabs to be able to approach, yet not so significant to cause turtle entrapment. Engineers and biologists theorized that the horseshoe crab would be unable to fully scale a fence that was past vertical and when they flipped or swam off, their orientation would send them back towards the bottom and not over the wall. Fence panels were set 15.24 cm into the seafloor to prevent burrowing underneath and resulted in an exposed height of 0.97 m above the seafloor (Fig 3). The prototype fence was marginally effective at preventing horseshoe crabs from passing the fence. The fence material, fence angle, and location created a corralling effect within the canal which prevented the accumulated horseshoe crabs from traveling out of the canal, against the current (Fig 2). The collection of horseshoe crabs and debris allowed them to bypass the fence and crawl over. While this was not consistently monitored, it was noted by divers during maintenance surveys. Due to the minimal reduction in horseshoe crab impingement, scouring under the prototype fence and high rates of seagrass fouling, it was determined that the permanent deterrent wall would need to be redesigned. The wall was positioned at the entrance of the intake canal to prevent the corralling effect and was angled with smooth panels to prevent the horseshoe crabs from crawling over, improving its effectiveness. The planning, permitting, and construction phase of the improved design required working closely with United States Fish and Wildlife Service, United States Army Corp of Engineers (USACE), Bervard County, EPA, FWC, and the FDEP. Permits required by the FDEP included a modification to the Conditions of Certification, an Environmental Resource Permit, and a Generic Permit for Stormwater Discharge from Large and Small Construction Activities. Four USACE permits were required; one for seagrass mitigation work, one for installation and one for removal of the prototype fence, and one for the installation of the permanent deterrent wall. Additionally, state-owned Submerged Lands Authorization was required to do the horseshoe crab deterrent and sea grass mitigation work, as well as negotiation of a Submerged Lands Lease with the State of Florida. All permits were issued by July 2017, and work was immediately commenced thereafter.

**Table 1:** Timeline of Horseshoe crab impingement mitigation strategies and monitoring activities at the Power Plant from November 1, 2014, to May 31, 2023.

Installation and Monitoring Events	Start	End
Prototype fence	11/1/2014	10/2017
Impinged horseshoe crab year-round monthly monitoring	4/10/2015	1/1/2019
Impinged horseshoe crabs tagged	6/29/2015	6/1/2016
Deterrent wall	11/2017	Present
Impinged horseshoe crab monitoring (January 1 - May 31)	1/1/2019	5/31/2023

### Permanent deterrent wall

**Fig 4:**
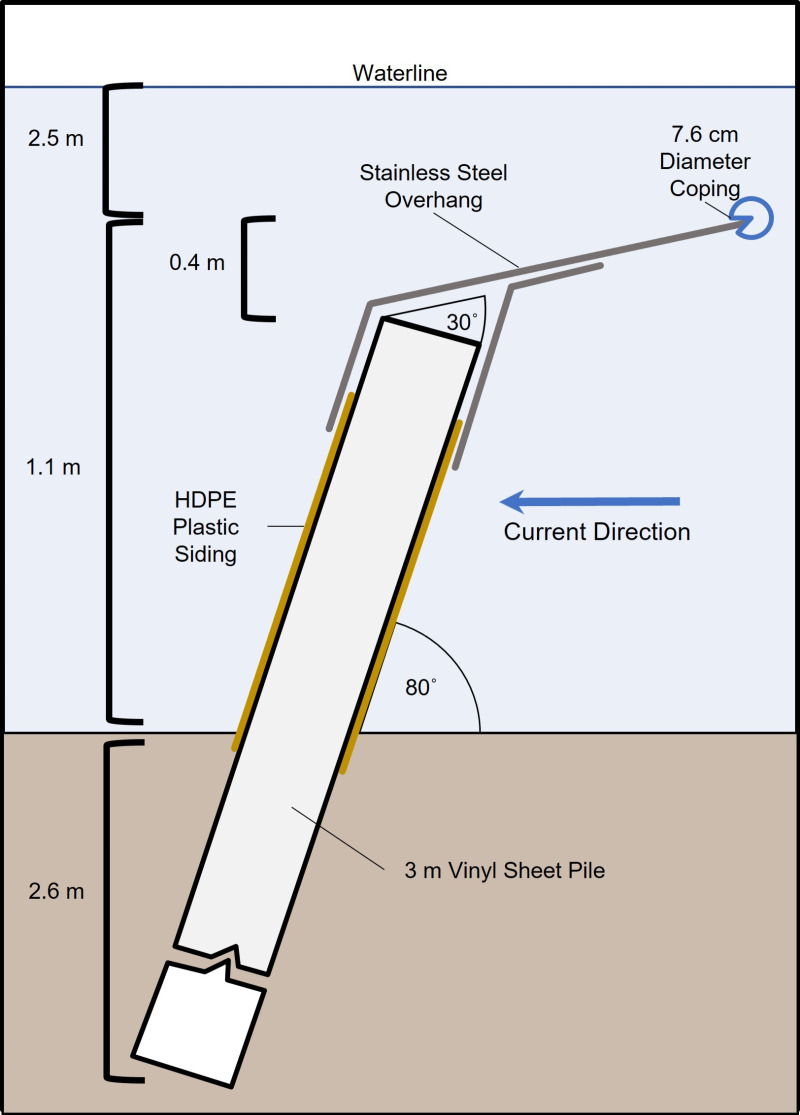
Schematic of the Deterrent Wall installed in the intake canal in November 2017.

The permanent deterrent wall was placed at an angle across the entrance of the intake canal to connect a seawall with the earthen peninsula (Fig 2). This angle was incorporated to direct horseshoe crab movements along the deterrent wall to areas away from the intake flow with reduced velocity, and to lessen the corralling effect observed at the previous location of the prototype fence. The construction of the deterrent wall began in July 2017 and was fully operational in November 2017. The materials selected for the deterrent wall had higher resistance to biofouling and greater life expectancy than the materials used for the prototype fence. The improved deterrent wall design consisted of a 3.05 m vinyl sheet pile coated with smooth high-density polyethylene sheets driven into the seafloor to a minimum depth of 2.57 m, and at an angle of 80° with the angle facing into the current. The minimum 2.57 m depth into the seafloor was used to prevent scouring and horseshoe crab burrowing under the wall. The total height of the permanent deterrent wall was 1.07 m above the seafloor, allowing 2.45 m of clearance over the top for fuel barges and other sea life (e.g., manatees, sea turtles) to pass freely. The total height included the sheet pile that was topped with a 0.41 m wide smooth stainless-steel plate oriented at an additional 30° angle and capped with a 7.62 cm diameter round coping (Fig 4). The 80° angle was sufficient to prevent horseshoe crabs from climbing over, but not significant enough to pose an entrapment hazard for sea turtles. This final design incorporated smooth surfaces and compound angles, intended to make it more difficult for biofouling to accumulate and to prevent the horseshoe crabs from gaining traction then deflecting them toward the riverbed to be carried downstream (Fig 4).

### Monitoring

From November 2014 through March 2015, after the prototype fence was installed, the total number of horseshoe crabs impinged was recorded. Beginning in April 2015, contractors hired by the plant began collecting biological data on the impinged horseshoe crabs. Three times per week (non-sequential), all debris, horseshoe crabs, and other animals were collected from the intake bar screens, placed in bins, data collected, and all living horseshoe crabs were released back into the water at designated locations. Between June 29, 2015, and June 30, 2016, horseshoe crabs collected during these surveys were tagged with a piece of embossing tape coded to the release location. This short-term tagging was used to determine whether the release locations were far enough from the intake channel to prevent animals from returning. Tagging methodology was based on Brockmann [22]. Thumb tacks with preprinted embossed labels were used to tag adult horseshoe crabs. Each tag was a unique color based on the release location north or south of the power plant. The horseshoe crabs were then transferred to release locations in shaded containers, where the date, release location, and injuries were recorded. If an animal was recaptured on the intake bar screens, the tag color and corresponding release location was documented to determine if there was a higher chance of return to the intake structures from either the north or south location. The contractors examined intake structures every 2–3 days and continued to record the number of horseshoe crabs impinged, their condition (alive or dead), and number of horseshoe crabs released. The mortality rate during this period is specific to horseshoe crabs’ condition when removed from the intake structure. Living horseshoe crabs were then transported to release points. In January 2019, monitoring data were reviewed with FWC, and the effectiveness of the wall was substantiated, after which, the monitoring effort was reduced to coincide with peak horseshoe crab activity in the Indian River Lagoon which occurs in four-day intervals between January 1 and May 31. Beginning in January 2020 per FWC approval, horseshoe crabs were removed from intake structures, transported and released only when the total count during a calendar month was over 250 horseshoe crabs. The mortality rate during this period reflects 100% mortality that occurs when all horseshoe crabs, alive or dead, are discarded along with seagrass and debris.

### Maintenance

To meet the requirements of the plant’s Conditions of Certification issued by FDEP, a Horseshoe Crab Deterrent Structure Maintenance and Monitoring Plan (MMP) was developed in coordination with the FWC. The plan identified structural maintenance activities, including quarterly inspections, repairs, debris removal of biofouling organisms, and reporting of such activities. During each inspection, divers documented any damage to the deterrent wall, biofouling that accumulated on the wall, and the number and location of horseshoe crabs at the wall or nearby shoreline. They also made repairs if feasible or recommended any necessary repairs.

### Analyses

Monitoring data were analyzed post implementation, to describe the overall effect of the prototype fence and deterrent wall on reducing horseshoe crab impingement. Specific data from April 10, 2015, to May 31, 2023, were compiled and analyzed in R version 4.2 (R Development Core Team 2021). A Welch’s T-test was performed to examine the difference in mean horseshoe crabs per collection, between intake collections, while the prototype fence and deterrent wall were present. The data were fit to a locally estimated scatterplot smoothing (loess) via the geom_smooth function in the ggplot2 package, to find the conditional mean and confidence interval of horseshoe crab counts.

Because count data were not collected until after the prototype fence was installed, data collected independently by FWC’s Fisheries Independent Monitoring (FIM) program and data collected by FWC’s Florida Horseshoe Crab Watch (FHCW) program were used to compare and observe horseshoe crab population trends in the NIRL. Florida Horseshoe Crab Watch data are collected by surveying known horseshoe crab spawning beaches during the highest tides; specific methods for surveys are outlined in Heres et al. [23], and the two closest survey sites within the NIRL were used for analysis (Fig 1). The FIM survey data collected in the NIRL was delineated as all surveys within the lagoon south of Latitude 29.7911908 and north of Latitude 27.8546390, the Brevard County line (Fig 1). Catch Per Unit Effort (CPUE) was calculated as horseshoe crabs per set using the methods outlined in Stevens et al. [24] and Winner et al. [25]. Gear types that do not catch horseshoe crabs were excluded from the analysis. The 21 m offshore seines composed of 3.2-mm knotless nylon mesh which covered 140 m^2^ per set, and 183 m haul seines composed of 183 m long, 38-mm knotted nylon mesh which covered 4120 m^2^ per set, were included in the analyses. Seine sampling sites were selected using monthly stratified-random sampling throughout the lagoon.

## Results

### Prototype fence, November 1, 2014 to October 31, 2017

The prototype fence was installed and maintained between November 1, 2014, and October 31, 2017, informally monitored by counting the total number of horseshoe crabs impinged, between November 1, 2014, and April 9, 2015, and formally monitored with routine collection events between April 10, 2015, and October 31, 2017. Informal monitoring was deemed insufficient when contractors made firsthand observation of horseshoe crabs climbing over the fence. As a result of collaboration with FWC and University of Florida, formal monitoring began on April 10, 2015. During the almost 3 years that the prototype fence was in place, 150,564 (average 50,188/year) horseshoe crabs were impinged, with a 12% mortality rate. These results and inspections confirmed that the design of the fence was not effective at preventing most horseshoe crabs from entering the intake system. Additionally, although the fence was inserted 15.24 cm into the seafloor, horseshoe crabs were able to burrow underneath it, where scouring at the fence bottom promoted sea floor material to wash out.

### Permanent deterrent wall

The permanent deterrent wall was installed in November 2017 and is still present at the time of publication. A total of 3,886 (average 647.7/year) horseshoe crabs were impinged with an intake system mortality rate of 19%, between November 2017 and June 2023. This was a 97.3% reduction in impingement, of horseshoe crabs after installation of the permanent deterrent wall. The change in deterrent location and the removal of the divider in the intake canal reduced the intake flow velocity from 0.46 mps to 0.23 mps. The average flow velocity where the permanent deterrent wall is installed is 0.12 mps (Fig 2). The location and significantly lower flow velocity made it easier for the horseshoe crabs to move downstream. The orientation of the deterrent wall was aligned with the water current, so as horseshoe crabs travelled from north to south, they naturally continued downstream along the wall thus returning to the lagoon rather than being corralled in the intake canal.

### Horseshoe crab monitoring

**Fig 5:**
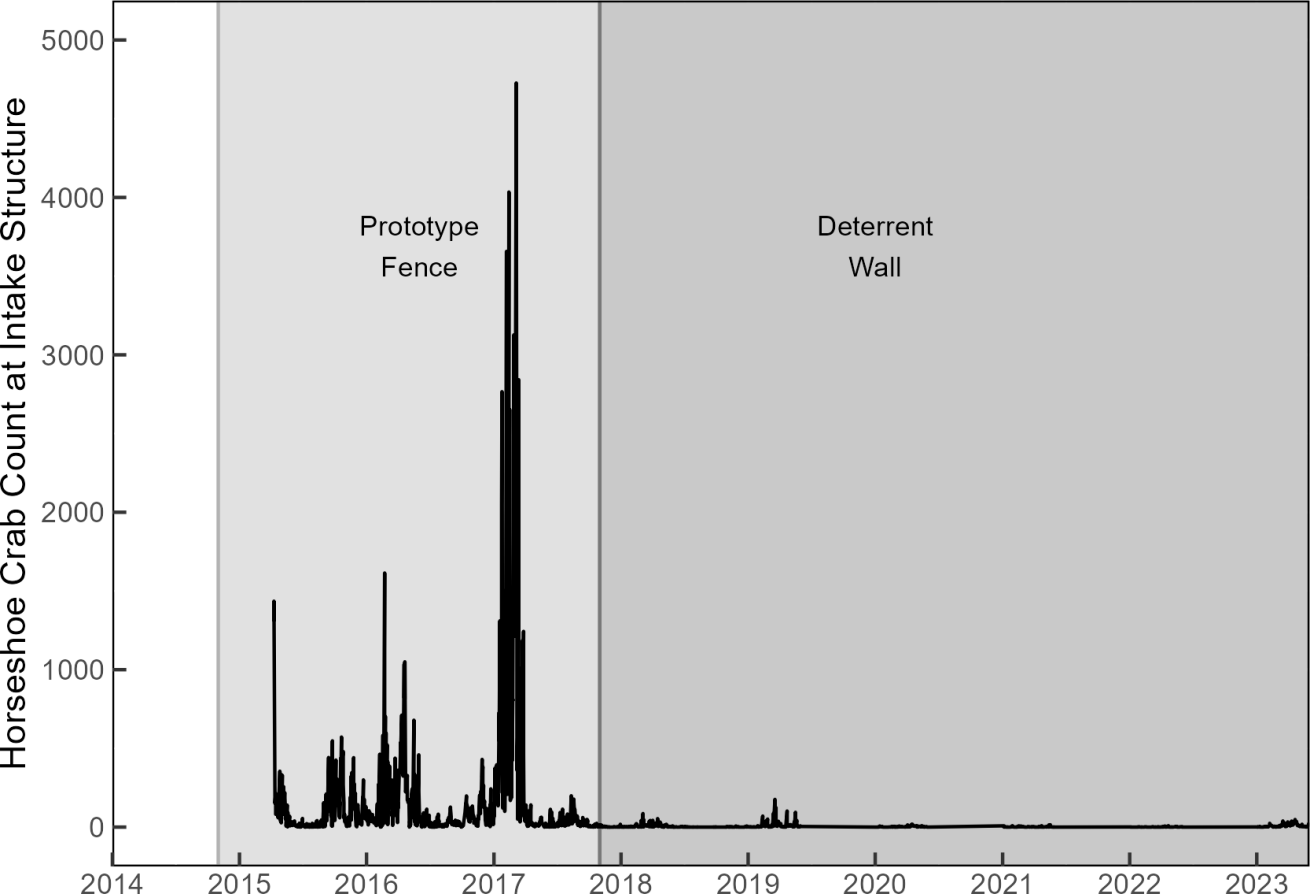
Number of horseshoe crabs counted and removed from the plant intake structure from April 10, 2015, to present. Shaded areas indicate when the Prototype Fence and the Deterrent wall were in place.

Prior to April 10, 2015, no formal monitoring of impinged Horseshoe crabs occurred (Table 1). Between April 10, 2015, and May 31, 2023, a total of 1,587 horseshoe crab collection events were performed by contractors in the intake debris collection systems. While the prototype fence was present, 814 collection events were conducted, and while the deterrent wall was present, 773 collection events were conducted. On average, 185 horseshoe crabs were collected at the intakes during each collection event while the prototype fence was in place. In contrast, an average of 5.2 horseshoe crabs were collected during each collection event when the deterrent wall was in place (Fig 5). The total number of horseshoe crabs counted, number of dead horseshoe crabs in the intake system, the fate of the horseshoe crabs after removal from intake system, and total death rate by year (intake mortality plus discard mortality) of each deterrent structure was recorded (Table 2). There was a significant difference between the mean number of horseshoe crabs per collection while the prototype fence was present (n = 814) versus the permanent deterrent wall being present (n = 773, t (814.3) = -11.01, p < 2.2e -16, Welch’s t-test).

**Table 2:** Total number of horseshoe crabs impinged at the plant intake bar screens, horseshoe crab mortality prior to return to natural habitat, the rate of mortality associated with impingement for each year of monitoring, and the fate of the horseshoe crabs after removal from the intake system. Monitoring began April 10, 2015, where live horseshoe crabs were collected, placed in ventilated plastic transport containers, and immediately transported by an enclosed vehicle to a FWC approved release location. Beginning January 2020 per FWC approval, horseshoe crabs were transported and released only when the total count during a calendar month was over 250 horseshoe crabs. The mortality rate during this period reflects 100% mortality that occurs when all horseshoe crabs, alive or dead, are discarded along with seagrass and debris.

Year	Structure Type	Total Intake System Count	Intake System Mortality	Fate of horseshoe crabs after removal from Intake System	Total Mortality Rate
**2016**	Prototype Fence	38,886	4,826	Released into NIRL	0.12
**2017**	Prototype Fence	87,226	6,145	Released into NIRL	0.07
**2018**	Deterrent Wall	1,304	106	Released into NIRL	0.08
**2019**	Deterrent Wall	1,309	192	Released into NIRL	0.15
**2020**	Deterrent Wall	231	68	Discarded	1.0
**2021**	Deterrent Wall	156	57	Discarded	1.0
**2022**	Deterrent Wall	63	31	Discarded	1.0
**2023**	Deterrent Wall	823	295	Discarded January, February, March, and May. Released into NIRL April.	0.75

**Fig 6:**
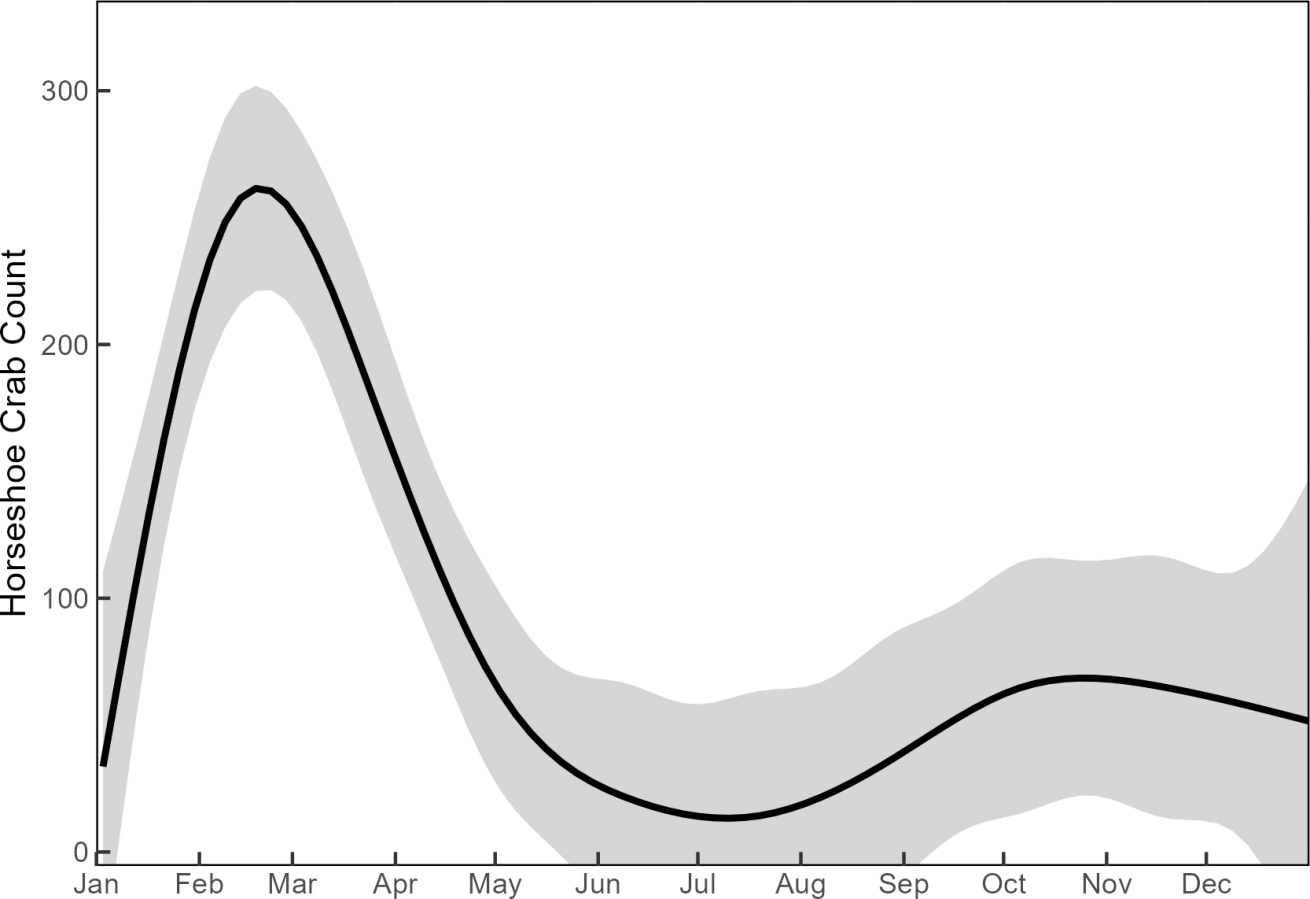
Loess curve fitted to horseshoe crabs counted and removed from the plant intake structure each month from November 11, 2014, to May 31, 2023. The conditional mean (solid line) and 0.95 confidence interval (shaded area) are displayed.

**Fig 7:**
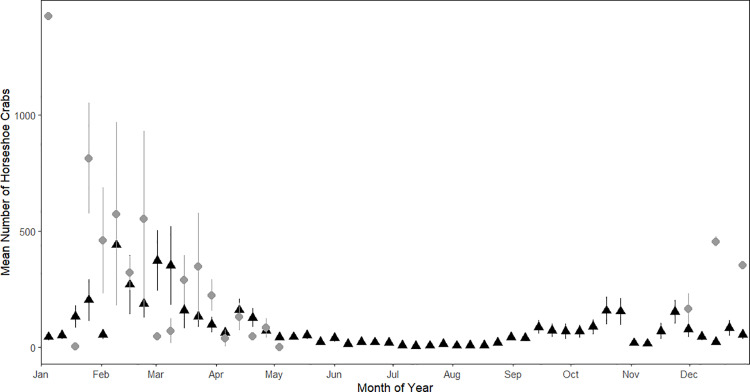
Mean number of horseshoe crabs and standard deviation (vertical lines) observed daily from April 10, 2015, to May 31, 2023, in the power plant intake system (gray circle) and during North Indian River Lagoon - Florida Horseshoe Crab Watch spawning surveys (black triangle). Observations were binned in weekly intervals.

A total of 5,260 horseshoe crabs were tagged during the debris removal from the intake structure screens, and 113 horseshoe crabs returned to the intake after previous capture and release (2.1%). Year-round monitoring revealed seasonal trends in impingement. A primary peak in seasonality of impingement between January and May was observed, with little activity during the summer months, and a comparatively smaller increase in activity in late fall, and early winter (Fig 6). This trend was also observed in the FHCW data (Fig 7).

### Maintenance

During 2018, eleven inspections were done on the deterrent wall. The most common damage to the deterrent wall was the loosening and failing of the water-resistant sheeting. This sheeting was found broken or peeled away from the wall, and the stainless-steel hardware was often corroded and needed replacement. On three occasions, sediment build up was documented on the south end of the wall. This build up was a concern because it reduced the height of the wall above the natural bottom, and the wall clearance for barge passage was reduced to 69 cm. Algae and barnacles were the primary fouling organisms and documented at ten of the eleven inspections. Algae fouling was described as light to moderate coverage in January and February, or after a cleaning, and moderate to heavy March through November. Similarly, barnacle fouling was light to moderate in January and February, or after a cleaning, and moderate to heavy the remaining inspections, especially July through October. An average of 22 horseshoe crabs were present at the wall during eleven of nineteen inspections, always on the east side of the wall (outside of the canal, and primarily at the south end). Additional concerns included buildup of drift algae, seaweed growth at the base of the wall, and the weight of biofouling that compromised the integrity of the deterrent wall.

Beginning in 2019, maintenance events consisted of four inspections conducted quarterly as required by the MMP. The MMP also required that an annual report documenting maintenance and monitoring activities for the calendar year be submitted to FWC and FDEP by December 15^th^ of each calendar year for review. Between 2019–2023, maintenance activities documented in these annual reports generally consisted of removing sediment buildup to maintain the original wall height and necessary clearance above the wall for manatees, sea turtles, and fuel barges. Additionally, the most persistent maintenance issue documented in the annual reports was biological fouling from algae, barnacles, and drift algae that required regular cleanings to prevent it from compromising the wall construction. In 2023, there was damage to the deterrent wall from storm events that caused the detachment of two HDPE sheets, and minor damage to two additional HDPE sheets. All four HDPE sheets were replaced, however, this contributed to an increase in impingement during 2023. Inspections between 2019–2023 showed that an average of 6.0 horseshoe crabs were present on the east side of the wall.

## Discussion

This project is an example of how innovative engineering, combined with biological knowledge, and regulatory engagement can unite to solve the decades old problem of horseshoe crab mortality in power plant intake systems. An unconventional approach was used to adaptively manage a project, with rapid response to observations, that facilitated the installation of the deterrent wall. These methods reduced the rate of horseshoe crab impingement by 97.3%, putting the plant in compliance with Section 316(b) of the Clean Water Act by exceeding the Environmental Protection Agency’s established 12-month impingement mortality performance standard reduction goal of 75% (CWA Section 316(b) [40 CFR 125.94 (c)(7)]).

Prior to the commissioning of the plant referenced in this study, a coal-powered plant was operating in the NIRL from 1965 to 2010. The coal powered plant was decommissioned and rebuilt as a natural gas plant in 2011. Between 1965 and 2011, the coal-powered plant functioned as an ecological sink for horseshoe crabs. Little information exists about the annual impingement rates in this plant’s intake systems, but one study from 1975 estimated that 69,662 horseshoe crabs were impinged annually [10], and a study from 1980 extrapolated that 39,097 horseshoe crabs were impinged annually [14]. Based on these estimates, the total loss of horseshoe crabs to impingement and discard of live animals, at the original coal-powered plant would be around 1.8 to 3.2 million horseshoe crabs, over the plant’s 46-year life span. There are several effective options for reducing horseshoe crab impingement mortality, ranging from hand removal of live horseshoe crabs directly off intake bar screens to constructing physical barriers to entry. The success of these prevention measures is dependent on the magnitude of the impingement, spawning seasonality, and overall cost. In this example the power plant chose a permanent deterrent wall to reduce labor of hand removal and significantly reduce the overall number of impinged horseshoe crabs.

The installation of the deterrent wall resulted in a reduction of impingement by 97.3%, when compared to pre-wall impingement. This success was achieved through collaboration and information sharing among plant staff, engineering consultants, and horseshoe crab scientists. The regular feedback resulted in rapid engineering modifications and implementation. While the prototype fence did not significantly reduce the number of crabs being impinged, it allowed engineers to ascertain that the location, water velocity, and angle of installation were key design elements. The prototype fence was located in a partially enclosed channel, where water velocity and fence angle contributed to corralling and concentrating horseshoe crabs. Additionally, the fabrication materials allowed horseshoe crabs to burrow under and climb over the prototype fence. Once this was understood, the prototype fence was removed, and the permanent deterrent wall built outside of the intake canal where water flow velocity was reduced, and horseshoe crabs could follow the shoreline away from the intake canal. The deterrent wall was inserted deeper into the seafloor than the previous fence to prevent scouring and gaps (Fig 2). The wall height of 0.97 m still allowed other organisms such as manatees and sea turtles to pass freely, and fuel barges to safely pass to and from the power plant dock inside the intake canal.

The deterrent wall is more robust compared to the prototype fence, composed of high-density polyethylene sheets and stainless-steel hardware that reduces risk of failure during storms and minimizes maintenance. While the deterrent wall is less likely to be damaged, the 2023 Maintenance and Monitoring Annual Report documented damage to the deterrent wall from storm events and this damage was attributed to an increase in impingement for the year (Table 2). The plant has established protocols for additional inspections after storm events, when feasible, to identify and repair any damage as soon as possible to maintain the effectiveness of the wall.

**Fig 8:**
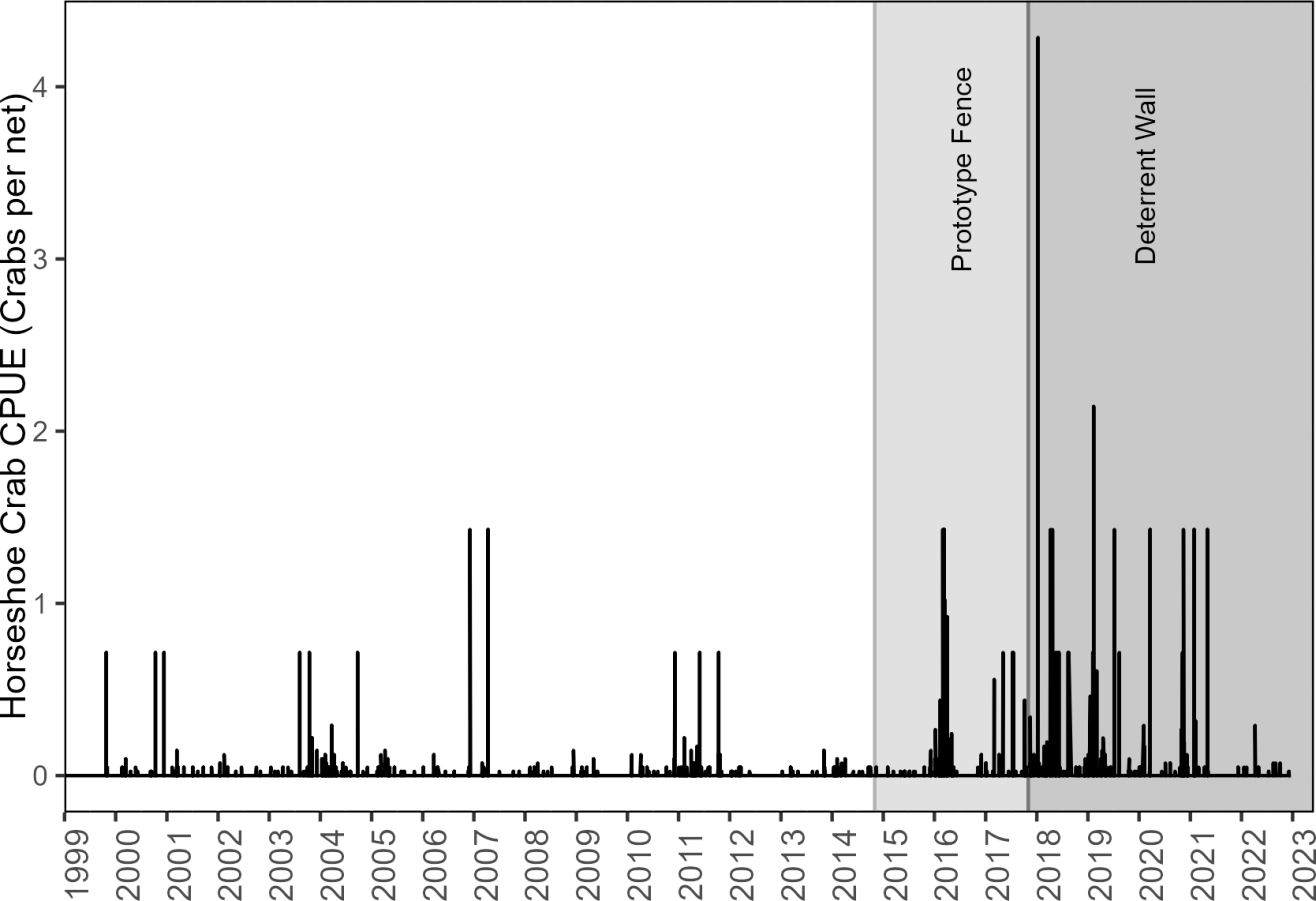
Number of horseshoe crabs caught per net (CPUE) in Brevard County during FWRI’s Fisheries Independent Monitoring sampling from 1999–2023. Shaded areas indicate the presence of the Prototype Fence and Deterrent Wall.

Horseshoe crab population monitoring in the NIRL is currently accomplished by the Florida Fish and Wildlife Research Institute through two data collection programs: 1) Fishery Independent Monitoring (FIM) multispecies trawls and seines and 2) beach spawning surveys by the Florida Horseshoe Crab Watch (FHCW). The horseshoe crab population in the NIRL has been characterized as having low densities and declining numbers, and there has been a concern for horseshoe crab conservation since the 1990s [5,10]. There are several possible explanations for the population decline, such as disease, increase in predator abundance, commercial harvest for the bait and aquarium trade, habitat destruction, and mortality due to impingement in power plant intake structures [4]. Recent time series data from FHCW (2016–2022) suggest the NIRL population have the highest densities of spawning horseshoe crabs of all the program’s monitored sites (FHCW unpublished data). Similarly, FIM surveys indicated a low but highly variable abundance of horseshoe crabs from 1999 through 2015, and a dramatic increase in adult horseshoe crabs after 2016 (FIM unpublished data) (Fig 8). The power plant data revealed that there was an increase in impingement at the power plant intake during the spawning season for horseshoe crabs in the NIRL (Fig 7). The concurrent timing of spawning and increased impingement occurs January through May (Fig 6). This suggests that horseshoe crabs are more likely to be impinged while searching for mates and spawning habitat, which is the only time in their life history where they exit the water along the shoreline. It is possible the increased velocity of the water as it flows into the plant may have disoriented the crabs because change in water pressure is a cue horseshoe crabs use to locate spawning habitat [26,27]. When the deterrent wall was erected, the water flow into the plant was reduced and horseshoe crabs no longer oriented towards the flow of water, thus sparing them from impingement. We postulate that the increase in horseshoe crabs during FIM surveys since 2016 in the NIRL is due to reduced mortality at the plant cooling water intake structures (Fig 8).

The complex engineering and biological challenges faced by this project are likely to be encountered at other power plants that impinge horseshoe crabs. It is the intent of this publication to be a tool for the managers of the 342 (U.S. Energy Information Administration) power plants in the horseshoe crab’s ecological range in the United States. Horseshoe crab impingement has been documented at power plants in several states, including New York, Maryland, Connecticut, and Florida. With no reporting requirement of impinged horseshoe crabs, there is no way to know the magnitude of the problem. We recommend that industry, state and federal governments, and conservation groups work cooperatively to determine the magnitude of the issue throughout the horseshoe crab’s range, so efficient and effective solutions can be implemented to reduce this avoidable mortality. Reduction of mortality associated with impingement would bolster efforts to improve horseshoe crab populations by the Atlantic States Marine Fisheries Commission, which manages the bait and biomedical industries but not mortality by power plants. Here we present early evidence of population increase in the NIRL horseshoe crab after installation of a deterrent wall at a power plant, demonstrating that power plants may have historically reduced the population in the region. Other plants within the horseshoe crab’s range may have similar effects on the surrounding population and impinge other valuable or endangered species, such as those 178 documented in the Kapar Power Station in Malasia [12]. Without cooperative monitoring and reporting it is impossible to know if similar remediation projects such as the one described in this study could be implemented to significantly benefit horseshoe crab populations and other threatened or endangered species.
